# Brownification increases winter mortality in fish

**DOI:** 10.1007/s00442-016-3779-y

**Published:** 2016-12-03

**Authors:** Per Hedström, David Bystedt, Jan Karlsson, Folmer Bokma, Pär Byström

**Affiliations:** Department of Ecology and Environmental Science, Umeå University, 90187 Umeå, Sweden

**Keywords:** Brownification, Winter mortality, Light limitation, Feeding efficiency, Metabolism

## Abstract

In northern climates, winter is a bottleneck for many organisms. Low light and resource availability constrains individual foraging rates, potentially leading to starvation and increased mortality. Increasing input of humic substances to aquatic ecosystems causes brownification of water and hence a further decrease of light availability, which may lead to further decreased foraging rates and starvation mortality during winter. To test this hypothesis, we measured the effects of experimentally increased humic water input on consumption and survival of young-of-the-year three-spined stickleback (*Gasterosteus aculeatus*) over winter in large outdoor enclosures. Population densities were estimated in autumn, and the following spring and food availability and consumption were monitored over winter. As hypothesized, mortality was higher under humic (76%) as compared to ambient conditions (64%). In addition, body condition and ingested prey biomass were lower under humic conditions, even though resource availability was not lower under humic conditions. Light conditions were significantly poorer under humic conditions. This suggests that increased mortality and decreased body condition and ingested prey biomass were not due to decreased resource availability but due to decreased search efficiency in this visual feeding consumer. Increased future brownification of aquatic systems may, therefore, negatively affect both recruitment and densities of fish.

**Electronic supplementary material:**

The online version of this article (doi:10.1007/s00442-016-3779-y) contains supplementary material, which is available to authorized users.

## Introduction

For animals living in a northern climate, the critical season is often the winter when low-temperature poor light conditions and low overall resource production constrain food intake and energy accumulation (Grøtan et al. 2005; Ultsch 2006; Helland et al. 2011). Consequently, starvation during winter is a major source of mortality in many organisms (Goss-Custard et al. 2001; Hurst 2007; McNamara and Houston 2008; Schröder 2013), with important effects on population dynamics, food web characteristics, and the geographical distribution of species (Helland et al. 2011; Humphries et al. 2002; Quayle et al. 2002; Shuter and Post 1990; Wootton 2007).

Although in ectothermic organisms, such as fish, low temperature decreases metabolism and energy demands (Elliot 1976; Jobling 1994, 2002), field studies in general show that winter starvation is an important source of mortality in small fish due to a low ratio of stored energy reserves to metabolic demands (Byström et al. 1998, 2006; Oliver et al. 1979; Schultz and Conover 1999). However, laboratory studies have shown that many species have the capacity to feed under winter conditions if food resources are abundant. Field studies also suggest that some species compete for resources and even grow under severe winter conditions (Post and Evans 1989; Biro et al. 2004; Finstad et al. 2004; Byström et al. 2006; Helland et al. 2011).

During winter, in northern climates, ice and snow cover reduces the amount of light that penetrates the water, which may decrease search efficiency for visually feeding aquatic consumers (Guthrie 1986; Shuter et al. 2012). Another factor decreasing light availability in recipient waters is the terrestrial export of coloured organic matter, an effect predicted to increase with climate change (Kokfelt et al. 2009; Larsen et al. 2011; Rosén 2005). Coloured organic matter causes brownification of water, which may further decrease search efficiency, and thereby consumption rates of visual consumers, such as fish (Estlander et al. 2012; Horppila et al. 2011; Jönsson et al. 2013). Thus, brownification may be particularly important during winter in an already light-limited environment, leading to increased starvation mortality over winter.

In this study, we used 16 YOY cohorts of three-spined sticklebacks (*Gasterosteus aculeatus*) in large outdoor experimental ponds to investigate, under natural conditions with ice and snow cover, if brownification due to increased input of humic water will cause higher winter mortality in young-of-the-year fish (YOY). More specifically, we measured (1) light conditions, (2) resource availability for sticklebacks, (3) stickleback diet and consumption, and (4) mortality rates and changes in body size distributions over a 7-month winter season. We hypothesize that brownification will result in decreased food consumption during winter in sticklebacks, which will, in turn, result in lower over-winter survival.

## Methods

### Experimental system

This study was performed at the Umeå University Experimental Ecosystem Facility (EXEF), a large-scale experimental pond system (73 m long, 23 m wide, with a depth of 1.6 m) divided into 20 enclosures (11.5 × 6.7 m) situated close to Umeå University, northern Sweden (63°48′N, 20°14′E). EXEF allows for long-term experimental ecosystem studies (i.e., spanning several years), including natural ice and snow cover during winter seasons. Each enclosure has separate water in- and outlets, and the facility allows for manipulation of input water chemistry and water temperature. Each enclosure contains a naturally functioning ecosystem with a benthic soft-bottom habitat as well as benthic and pelagic primary producers, invertebrate consumers and an introduced fish top consumer, and three-spined stickleback. The present long-term study at EXEF was initiated in May 2012 and three-spined sticklebacks were introduced the 22nd of May with the aim to study top consumer and whole-ecosystem responses to climate change with increased temperature (‘Warm’) and humic water input (‘Humic’) as experimental manipulations in a factorial design. The introduction consisted of 40 adult three-spined sticklebacks to each enclosure collected during spawning migration into a shallow coastal spawning bay in the Bothnian Sea (63°45′14″N, 20°32′18″E).

In 2012, during the ice-free season, from May to October, eight enclosures were heated (3 °C above ambient temperature), while eight were at ambient temperature. Each ‘Warm’ enclosure was heated with individual heat exchangers, where water slowly circulated through the heat exchangers and back to each enclosure. From May to October, four heated and four ambient enclosures received a continuous input of natural humic water (‘Humic’) (Table 1) collected from a mid-sized boreal stream, Pålböleån, located 20 km North East of EXEF (63°48′N, 20°14′E) to increase water colour and dissolved organic carbon (DOC) concentrations and thereby experimentally induce climate-change brownification of freshwater ecosystems. The water for the Humic treatment was collected and transported weekly from the source stream in a tanker truck to the EXEF facility and kept in a 40 m^3^ tank. Humic water was then continuously distributed to each of the eight enclosures at a rate of 4 L min^−1^. The other eight enclosures received equal rates of clear water (Table 1) from the Umeå municipality groundwater source. When ice started to form in October in ambient temperature enclosures, both heating and humic (and clear) water additions were terminated for the season. Temperature differences in enclosure disappeared within a week, whereas the DOC concentration difference was present throughout the winter season. Hence, we were able to study the direct effects of increased humic input (i.e., brownification) on the survival from autumn over winter of the YOY cohorts that the introduced adult stickleback produced in spring 2012. Treatment effects during the ice-free growth season 2012 on YOY performance and recruitment levels and the long-term response of stickleback populations and ecosystems have been and will be reported elsewhere (Jonsson et al. 2015; Rodríguez et al. 2016; Hedström et al. 2016 in submitted).

**Table 1:** Average (±SE) values of water chemistry of input water during 2012

	Groundwater source^a^	Pålböleån^b^ (humic treatment origin)
pH	8.05 ± 0.09	6.55 ± 0.08
Conductivity (mS m^−1^)	12 ± 0	3.4 ± 0.19
Ca (mg L^−1^)	18.5 ± 0.66	4.7 ± 0.32
K (mg L^−1^)	1.8 ± 0.08	4.4 ± 0.04
Mg (mg L^−1^)	1.39 ± 0.08	0.46 ± 0.05
Na (mg L^−1^)	1.79 ± 0.08	1.33 ± 0.16
DOC^c^ (mg L^−1^)	1.06 ± 0.03	22.5 ± 1.9
N^c^ (μg L^−1^)	69 ± 4.6	622 ± 23.2
P^c^ (μg L^−1^)	3.8 ± 1.3	72.4 ± 15.8

^a^Data from the municipality of Umeå. For further information: http://www.vakin.se

^b^Pålböleån: data from the monitoring program of running waters in Västerbotten. County board samples are collected at 63°54′38.35″N and 20°34′9.02″E. For further information: lansstyrelsen.se

^c^Samples analysed at Umeå University

### Abiotic condition sampling

Water temperature (°C) and light intensity [PAR, photosynthetic available radiation (μmol/m^2^/s)] were continuously measured at 0.8 m water depth with temperature sensors (TH2-F, UMS Germany) and light sensors (SQ-110, Apogee USA) and, averaged over 15 min, recorded with loggers (Delta-T Devices, UK). Temperature and light intensity were calculated as monthly averages of daily noon (12.00 p.m.) values. Due to logger failure, there are missing data points from a number of enclosures between 4th February and 11th March. However, this does not have any major implications for the results of this study due to the stable abiotic conditions in the enclosures during this part of the winter. The ice cover thickness (cm) and the concentration of dissolved organic carbon (DOC) and oxygen concentration were measured at six occasions between 28 January and 16 April. For DOC analysis, water was filtered through burnt (550 °C, 1 h) 0.45 μm GF/F filters to 50 ml Falcon tubes, acidified with 500 μl 1.2 M HCl and stored cold (4°C), until analysed with a combustion chamber (IL-550 TOC-TN analyser, Hach Lange Gmbh). Oxygen concentrations were estimated in situ using an oxygen-temperature meter (ProODO, YSI Inc.)

### Invertebrate resource sampling

Prior to winter, macroinvertebrates were initially sampled on 24 September and zooplankton on 22 October 2012. During ice-covered season, resources were sampled on the same occasions as winter sampling of fish in 2013 (see below). Macroinvertebrates were sampled with a net (30 cm wide, 1 mm mesh size). In each enclosure, the net was drawn at the bottom substrate for a distance of 60 cm in October 2012 and 30 cm during the ice cover period in 2013. Each sample was then preserved in ethanol for later analysis. Zooplankton was sampled with a zooplankton net (diameter 20 cm, 100 µm mesh size) drawn 1.4 m vertically in each enclosure in both summer and winter. The samples were preserved in Lugol’s solution for later analysis. In the laboratory, macroinvertebrates and copepods from the benthic samples were classified into order, family, or genus, counted, and measured: their length was transformed to obtain dry biomass (mg) using length-weight regressions (macroinvertebrates; Persson et al. 1996 and references therein and copepods Botrell et al. 1976). Zooplankton was classified into family, counted and length measured to obtain dry biomass (µg) with length-weight regressions (Botrell et al. 1976; Dumont et al. 1975). As only a minor share of the species in the benthic fauna made a contribution to the actual diet in sticklebacks, resource levels were estimated on species groups relevant to sticklebacks, namely, zooplankton, chironomidae, and ephemeroptera.

### Fish sampling

We estimated YOY numbers and adults by seine-netting each enclosure with three sequential hauls with a seine net 11–13 October 2012 and 7–9 May 2013 (9 days after ice off). The seine net (mesh size in the fish bag 1.5 mm) was specially designed to match depth and width the size of the enclosures. All captured fish were photographed from above and, after the third seine-netting effort, released back into the enclosure. Length of the fish was estimated from photographs using an image analysis software developed at the department specifically for this purpose. The software gives the relative length of objects, which is transformed to fish length using a reference plate of known length in each photo. Population densities in each enclosure were calculated by the three-pass removal method (Zippin 1956).

### Diet analyses

A subsample of 30 YOY fish from each enclosure from the October sampling was deep-frozen for later analysis. During winter, ten YOY fish (if possible) were sampled from each pond from the ice with a landing net at three occasions: 28 January–1 February, 25 February–1 March, and 1–5 April in 2013. Because of difficulties to obtain ten individuals at the second sampling occasion, a minimum of five fish were caught except in one enclosure, where only two fish were captured. Captured fish were frozen for later analysis. In the laboratory, fish were length measured to nearest 0.5 mm and wet weight to nearest 1 mg. Stomach content was analysed and classified equally as for resources, counted and length measured to obtain dry biomass (mg) of consumed prey with length-weight regressions for zooplankton and macroinvertebrates (Dumont et al. 1975; Botrell et al. 1976; Persson et al. 1996 and references therein). To standardize prey consumption of individual fish, the prey dry biomass was divided with the wet weight of the fish. For body condition comparison, we used the ratio wet body mass (mg)/body length (mm)^3^ × 100.

### Winter mortality

Mortality over winter was calculated as

$${\text{Mortality rate}} = \frac{{\ln \left( {N_{1} } \right) - { \ln }\left( {N_{2} } \right)}}{t} ,$$

where *N*_1_ is autumn population size, *N*_2_ is spring population size, and *t* is number of days between population estimates in autumn and the following spring. To analyse if winter mortality was size dependent, we used Bayesian statistics to model population size development from autumn to spring with respect to body length (Online Resource 1).

The design of the ongoing experiment, sampling methods, collection of experimental fish, and method of sacrifices in this study comply with the current laws of Sweden and were approved by the Animal Review Board at the Court of Appeal of Northern Norrland in Umeå (CFN, License No. A-24-11 to Pär Byström).

### Statistical approach and analyses

The initial population densities of YOY stickleback in October 2012 varied substantially. This was due to strong negative effects of temperature treatment on YOY stickleback performance and density during the summer season (average YOY density ambient: 1689 ± 164, humic: 1983 ± 247, warm: 1041 ± 36, warm × humic: 1035 ± 79, average length ambient: 20.5 ± 0.2, humic: 20.4 ± 0.9, warm: 20.5 ± 1.0, warm × humic: 20.2 ± 0.4 average ±1 SE), (Hedström et al. 2016 in submitted). However, heating was terminated in October and no main or interaction effects of temperature treatment were statistically found for any of the biotic response variables analysed in this study, part form a negative effect of past heating on chironomidae abundance during winter (full models, including temperature effects and model selection, see Online Resource 2). We, therefore, assume that the temperature treatment had minor if any effect on our results and collapsed the temperature treatments in this study to, respectively, ambient (clear water) and humic treatment only, with eight replicates each. In addition, as number of the introduced adults in spring that survived to autumn sampling was low, 1.5 ± 0.35 and 2.5 ± 0.5 (average ±1 SE) in ambient and humic treatments, respectively, we assumed that adult impact on YOY performance over winter to be minor. For physical and chemical data (i.e., temperature, light intensity, DOC, and ice cover) and biotic data (i.e., diet composition and ingested prey biomass, resource abundance of zooplankton chironomidae, and total macroinvertebrate biomass), we used a linear mixed-effect model with Humic and Time as fixed factors and enclosure as random factor (Pinheiro et al. 2014). To analyse differences in size-dependent winter mortality patterns between treatments, we used multi variate analysis of variance (MANOVA) on the characteristics of the survival curve (Online Resource 1) retained in the Bayesian modelling output (i.e., slope at inflection point and body length at inflection point). Data were log-transformed when necessary to meet distribution and homogeneity assumptions. Proportional data (i.e., diet composition) were logit transformed (Warton and Hui 2011).

## Results

**Fig. 1:**
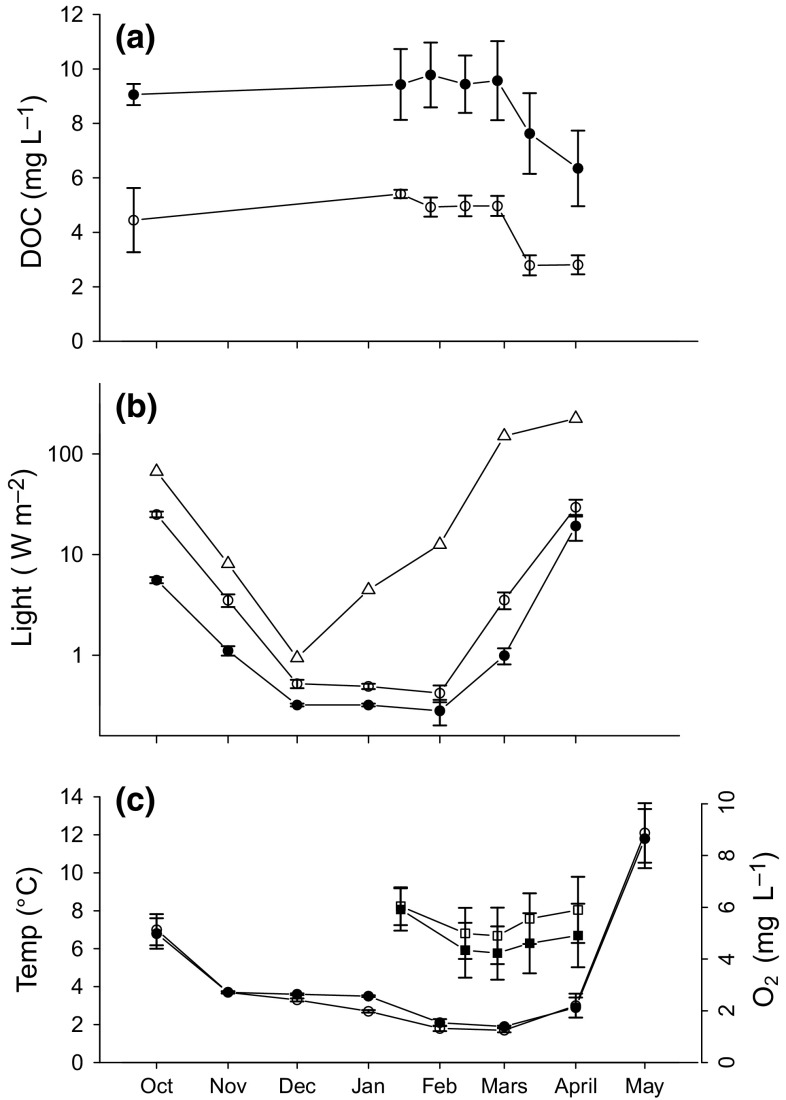
**a** Average DOC levels over time in ambient and humic enclosures, *open circles* are ambient treatment, *closed circles* are humic treatment, **b** *light* availability over time in the water column in ambient and humic enclosures and incoming surface light, *triangles* are incoming daylight, *open circles* are ambient treatment, *closed circles* are humic treatment. **c** Average temperature (*left y-axis*) and oxygen concentration (*right y-axis*) over time in ambient and humic enclosures, *open circles* are temperature in ambient treatment, *closed circles* are temperature in humic treatment, *open squares* are oxygen concentration in ambient treatment, *closed squares* are average oxygen concentration in humic treatment. *Error bars* denote ±1 SE

Enclosures were covered by ice from October 28 in 2012 to April 29 in 2013, apart from 1 week from November 18 to 25. Average ice thickness (±1 SD) was 40.5 ± 3.8 and 40.1 ± 3.2 cm in ambient and humic enclosures, respectively, based on late January to mid April estimates, and did not differ significantly between treatments (Table 2). DOC level decreased over time, but was approximately two times higher in humic treatment compared to ambient throughout the winter (Table 2; Fig. 1a). Light intensity at 0.8 m depth under the ice varied over time, with the lowest values during December through February despite increasing incoming light, and was lower in humic compared to ambient enclosures (Table 2; Fig. 1b). Temperature and oxygen concentrations did not differ between ambient and humic enclosures over the winter season (Table 2; Fig. 1c).

**Table 2:** Analysis of variance (*F* values) of the linear mixed-effect model on DOC, light, temperature, oxygen, and ice thickness

Source of variation	*df*	DOC	*df*	Light	Temp	*df*	Oxygen	Ice thickness
Humic	1, 14	21.99***	1, 14	12.56**	0.001	1, 14	0.32	0.129
Time	7, 98	19.2***	6, 80	22.45***	27.18***	5, 70	29.9 ***	8.21***
Humic × time	7, 98	1.46	6, 80	4.58***	0.30	5, 70	0.08	0.35

Significances levels: * <0.05, ** <0.01, and *** <0.001

**Fig. 2:**
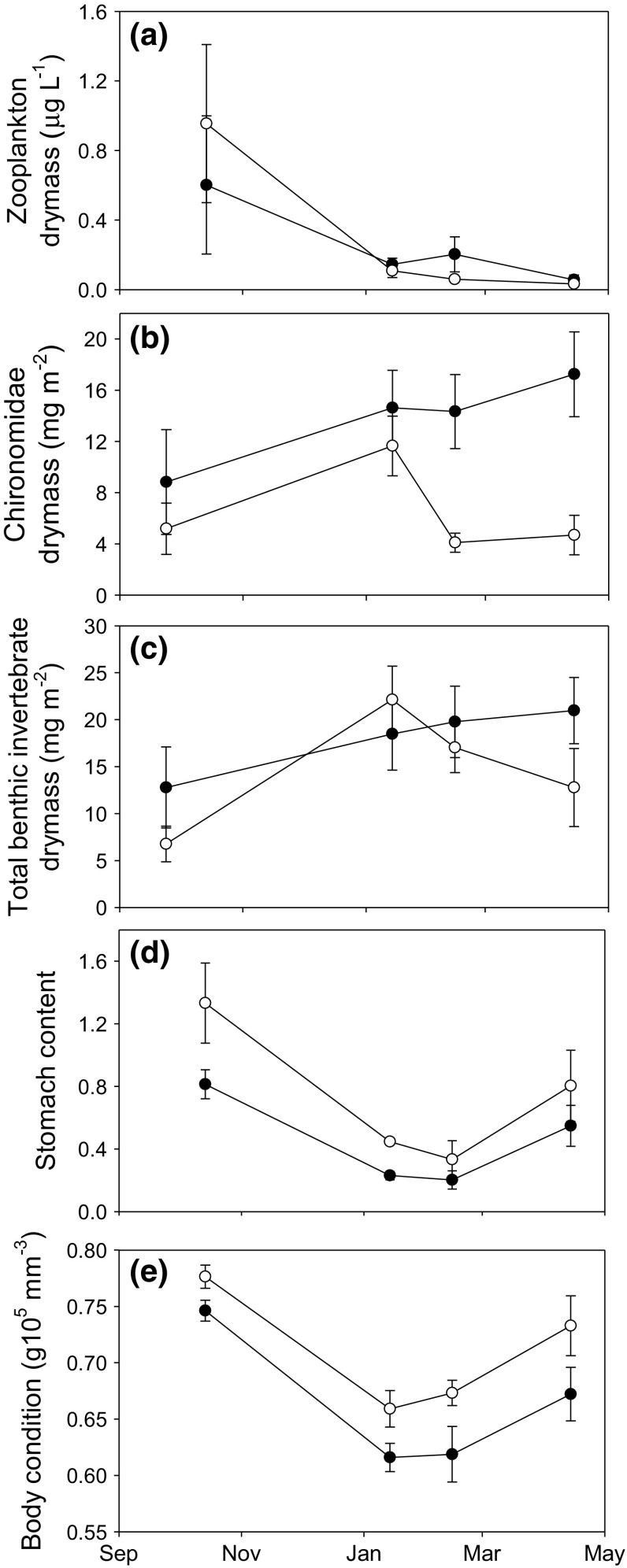
Average dry mass of **a** *Zooplankton*, **b** *Chironomids,* and **c** *Total* macro invertebrates over time in humic and ambient enclosures. **d** Average total prey biomass (dry weight per wet weight of fish) in stickleback stomachs and **e** *body* condition of sticklebacks over time in ambient and humic enclosures. *Open circles* are ambient treatment, *closed circles* are humic treatment, *error bars* denote ±1 SE

Zooplankton biomass decreased over time but did not differ significantly between treatments (Table 3; Fig. 2a). Chironomid biomass was lower in ambient enclosures, whereas total biomass of macroinvertebrates did not differ between treatments but changed over time (Table 3; Fig. 2b, c).

**Table 3:** Analysis of variance (*F* values) of the linear mixed-effect model with humic treatment and time as fixed factors on resources (total resources, chironomids, and zooplankton), diet, and body condition of fish

Source of variation	*df*	Resources				Fish	
		Zooplankton		Chironomids	Total	Ingested biomass	Body condition
Humic	1, 14	0.001	5.54*		0.45	5.12*	8.03*
Time	1, 46	17.8***	1.42		5.30*	5.18*	4.96*
Humic × time	1, 46	1.55	3.06^**+**^		0.52	0.62	0.41

Significance levels: ^+^ <0.1, * <0.05, ** <0.01, and *** <0.001

The biomass of ingested prey by sticklebacks was higher in ambient compared to humic enclosures during the whole winter (Table 3; Fig. 2d). Ingested prey biomass decreased initially and increased again towards the end of the ice-covered period (Table 3; Fig. 2d). Body condition of sticklebacks was higher in ambient compared to humic enclosures (Table 3; Fig. 2e).

**Fig. 3:**
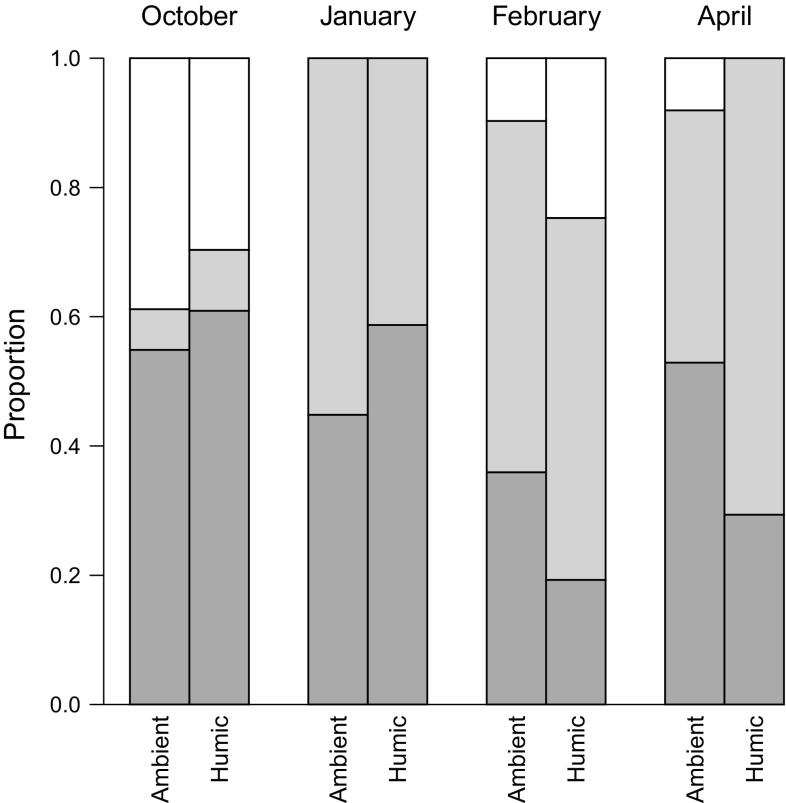
Diets (proportion of total biomass) of sticklebacks in ambient and humic enclosures over winter. From below: relative contribution of chironomidae (*dark grey*) zooplankton (*light grey*), other specimen (*white*) in diet

Chironomids and zooplankton (mainly copepods) dominated the diets and there was no treatment effect on diet composition (Fig. 3, analysis of variance on linear mixed-effect model, *F*_2,28_ < 1.7, *P* > 0.21).

**Fig. 4:**
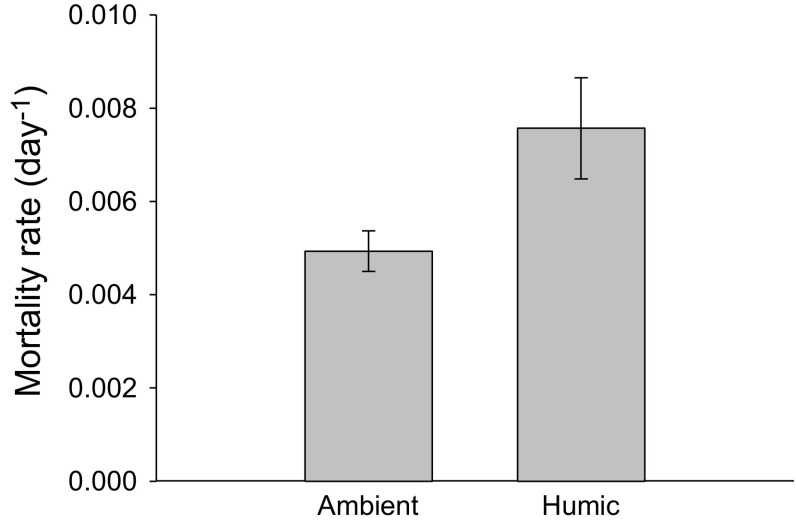
Average mortality rates of sticklebacks over winter in ambient and humic enclosures. *Error bars* denote ±1 SE

Mortality rate of sticklebacks over winter was higher in humic compared to ambient treatment (Fig. 4, ANOVA, *F*_1,14_ = 4.4, *P* = 0.05), which corresponds to a mortality of 76% in humic enclosures and 64% in ambient enclosures.

**Fig. 5:**
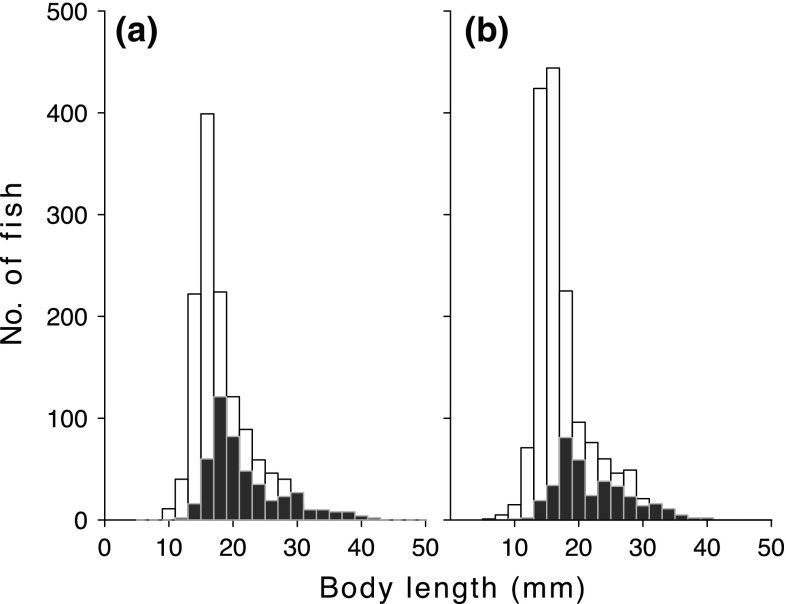
Average size distribution of sticklebacks in autumn (*open bars*) and the following spring (*closed bars*) for **a** ambient and **b** humic enclosures

Overall, winter mortality was negatively size dependent (Online Resource 1, see also Fig. 5), and parameters of size dependency did not differ between treatments (MANOVA for parameter values in the survival function; length at inflection and slope at inflection: *F*_1,14_ = 0.17, *P* = 0.85, Online Resource 1). There were no differences in autumn or the following spring between treatments in average size or distribution measures (skewness and median) (ANOVA on linear mixed-effect model, *F*_1,14_ = 1.53–2.44, *P* = 0.14–0.24, Fig. 5).

## Discussion

The results from this study show that brownification due to increased humic water input had negative effects on performance and survival of three-spined sticklebacks over winter. Sticklebacks in the humic treatment had a significantly lower ingested prey biomass and lower body condition compared to fish kept under ambient conditions. This lower ingested prey biomass in the humic enclosures was not a result of lower resource availability: resource availability was similar (zooplankton) or even higher (chironomid) than under ambient conditions. This suggests that decreased food intake was due to the observed poorer light conditions under humic conditions. Oxygen deficiency was probably not the cause of the observed differences, because oxygen concentrations did not differ between ambient and humic treatments. In addition, temperature, which may constrain foraging capacity in fish (Wootton 1990; Jobling 1994), did not differ between treatments. Taken together, this suggests that the change in light conditions during winter was the main factor causing differences in survival between treatments.

The presence of a negative size-dependent winter mortality due to starvation will hold true if individuals are not able to feed over winter (Oliver et al. 1979; Post and Evans 1989). Although both search efficiency and digestion capacity decrease with decreasing temperature, many temperate fish species, including three-spined sticklebacks, have the ability to feed at low water temperatures under laboratory conditions (Post and Evans 1989; Lefébure et al. 2011). Still, negative size correlation to winter mortality has been shown in most of these species in natural systems (Toneys and Coble 1979; Post and Evans 1989; Byström et al. 1998; Biro et al. 2004). Both decreasing resource production and impoverished light conditions have been suggested to be the main reasons for observed starvation mortality over winter in young fish (Byström et al. 2006). The Bayesian modelling approach in our study showed that mortality was in general negatively size dependent, even though no differences could be detected between treatments. Hence, the presence of negative size selective mortality in combination with the lower stomach content and decreased body condition strongly suggests that starvation was the main mechanism behind the observed mortality over winter.

Poor light conditions affect foraging rates negatively by decreasing capacities, such as reactive distance and capture success (Vogel and Beauchamp 1999; Helland et al. 2011; Ulvan et al. 2012; Jönsson et al. 2013). Similarly, as humic substances negatively affect light conditions, this should impair vision for visual predators, although experimental evidence for negative effects on fish foraging rates by brownification is contradictory and species specific (Estlander et al. 2012; Jönsson et al. 2013; Nurminen et al. 2014) and brownification has been suggested to cause changes in species interactions and affect competitive interaction between fish (Helland et al. 2011; Ulvan et al. 2012; Stasko et al. 2015). Light availability in our study seemed to affect consumption rates in stickleback as prey biomass in stomachs was overall lower at midwinter conditions with very low light intensities compared to early spring conditions with higher light intensities, despite similar temperatures and lower or similar resource abundance. More importantly, the experimentally induced brownification likely caused a further reduction in consumption rates as indicated by the lower prey biomass in stomachs and a lower body condition in stickleback in the humic treatment. Still, arguments could be raised that the natural humic water used in our study contains, e.g., toxic chemical compounds that caused the observed negative effect on the performance of YOY sticklebacks instead of our suggested main mechanism of impaired light conditions through brownification. However, several lines of counterarguments could be put forward to refute this hypothesis. First, there were no negative effects of humic treatments on YOY performance or densities during the summer 2012 (see methods, Hedström et al. 2016 in submitted). Second, there were no negative effects of humic treatments on other organisms during winter in this study. Finally, the fish community in the river from which the humic water is collected includes salmonid species, like such as trout (*Salmo trutta*)(County Administrative Board of Västerbotten 2015) which is regarded to be a sensitive species to poor water quality (Alabaster and Lloyd 1982). Thus, our results strongly suggest that when fish are able to feed during winter, increased humic concentrations and brownification negatively affect the feeding abilities which in turn may cause increased winter mortality in fish.

The climate change induced increase of humic substances in recipient aquatic systems also has profound effects during the ice-free period. Even though terrestrial organic matter can support the growth of individual consumers, it appears to reduce rather than increase whole lake secondary production (Kelly et al. 2014; Karlsson et al. 2015). Thus, the effects of increased humic levels acts over the whole year but in different ways depending on season, i.e., in winter by affecting the feeding success and survival of especially YOY fish, and in summer largely through lower primary production and resource supply to fish (Craig et al. 2015; Karlsson et al. 2015; Seekell et al. 2015).

However, evolutionary responses to increased brownification may counteract the negative effects on foraging rates as fish may evolve larger eyes and/or increased photoreceptor density (Fontanier and Tobler 2009; Dugas and Franssen 2012). Still, brownification not only decrease intake rates but may also strengthen the negative effects on fish performance further by decreasing benthic resource densities during winter. This is because negative effects of increased humic levels during summer are especially pronounced on benthic primary productions and invertebrate production (Karlsson et al. 2009; Craig et al. 2015), i.e., the main resource for fish during winter (Byström et al. 2006). Hence, future climate-change affects both productivity during summer and survival of YOY fish during winter. Therefore, it is likely that future production and biomass of many fish populations will be lower than what is present today.

## Electronic supplementary material

Below is the link to the electronic supplementary material.

Supplementary material 1 (DOCX 552 kb)

Supplementary material 2 (DOCX 44 kb)
