# Supplementary material for: Brownification increases winter mortality in fish
Source: Oecologia. 2016 Dec 3;183(2):587–95. doi: 10.1007/s00442-016-3779-y (PMC5306166; doi:10.1007/s00442-016-3779-y)
Supplement: Supplementary file 1 — Supplementary material 1 (DOCX 552 kb) [file 442_2016_3779_MOESM1_ESM.docx]

BROWNIFICATION INCREASES WINTER MORTALITY IN FISH

Running head: BROWNIFICATION INCREASES WINTER MORTALITY

Electronic Supplementary Material 1

Per Hedström*, David Bystedt, Jan Karlsson, Folmer Bokma and Pär Byström

Department of Ecology and Environmental science, Umeå University, Umeå, SE 90187, Sweden

*Corresponding author: [per.hedstrom@umu.se](mailto:per.hedstrom@umu.se)

Keywords: Brownification, winter mortality, light limitation, feeding efficiency, metabolism

Primary research article

BROWNIFICATION INCREASES WINTER MORTALITY IN FISH

Running head: BROWNIFICATION INCREASES WINTER MORTALITY

Electronic Supplementary Material 1

Per Hedström*, David Bystedt, Jan Karlsson, Folmer Bokma and Pär Byström

Department of Ecology and Environmental science, Umeå University, Umeå, SE 90187, Sweden

*Corresponding author: [per.hedstrom@umu.se](mailto:per.hedstrom@umu.se), Phone: +46706034448

Keywords: Brownification, winter mortality, light limitation, feeding efficiency, metabolism

Primary research article

**Estimation of size-dependent survival over winter**

To analyse whether winter mortality was size-dependent, we used Bayesian statistics to model change in the number of individuals in a population size and their body length distribution from autumn to spring. Before winter, a population in an enclosure consists of *n_b_* individuals, of which *n_a_* survive until after winter. Survival probability *P_s_* depends on body length *l_b_* as:

Eq. S1: $P_{s}=\frac{1}{1+\exp\left(c_{1}\left(l_{b}-c_{2}\right)\right)}$

In this (logistic) survival curve, parameter *c*_1_ indicates whether survival probability increases (*c*_1_<0) or decreases (*c*_1_>0) with body length, and parameter *c*_2_ is the length at which *P_s_*=0.5. During winter individuals may grow, and growth (*l_a_*-*l_b_*) is assumed to be exponentially distributed with mean *γ*:

Eq. S2: $l_{a}-l_{b}=g\propto E(\gamma)$

Thus, we assume that individuals do not shrink over winter (since the domain of the exponential distribution is positive). However, we assume that body length is measured with normally distributed error so that:

Eq. S3: $l_{a}-\left(l_{b}+g\right)\propto N(0,\sigma)$

If all individuals would have been measured before and after winter, and if they were individually recognizable, it would be easy to estimate growth (*g*) as *l_a_*-*l_b_*, and parameters *c*_1_ and *c*_2_ by logistic regression. However, only *n_b,m_* out of *n_b_* and *n_a,m_* out of *n_a_* individuals were measured before and after winter, respectively and they are not individually recognizable. Consequently, we do not know which of the individuals measured before winter, were measured again after winter. In addition, we do not know the body lengths of the *n_b_*-*n_b,m_* and *n_a_*-*n_a,m_* individuals that were not measured. Therefore, we used a Bayesian algorithm for parameter estimation.

The parameters of the model are the lengths of all individuals before and after winter, their growth, and parameters *c*_1_ and *c*_2_ (Eq. S1), *γ* (Eq. S2) and *σ* (Eq. S3). Also, there is a parameter *z* that identifies each of the *n_a_* surviving individuals as one of the *n_b_* individuals that were alive before winter. In other words, *z* contains information on “who-is-who”, and thereby indicates which individuals survived winter. The model is illustrated in figure S1.

Parameters were assigned the following prior distributions: the lengths of the *n_b_*-*n_b,m_* individuals that were not measured before winter were assumed to follow the same length distribution as the *n_b,m_* individuals that were measured. Similarly, the lengths of the *n_a_*-*n_a,m_* surviving individuals that were not measured after winter were assumed to follow the same distribution as the lengths of the *n_a,m_* individuals that were measured after winter. (This is appropriate because most individuals were measured.) To avoid assuming a particular functional form for these distributions, they were determined by kernel density estimation. Thus, the prior probability that an individual not measured before winter has length *l* is proportional to the kernel density of the length measurements *l_b_*, evaluated at *l*. Survival function parameters *c*_1_ and *c*_2_ were assigned uniform priors <∞,∞>, and parameters *γ* (mean growth) and *σ* (measurement error) uniform priors [0,∞>. Finally, for “who-is-who”parameter *z*, all correspondences between individuals before and after winter were considered *a priori* equally likely.


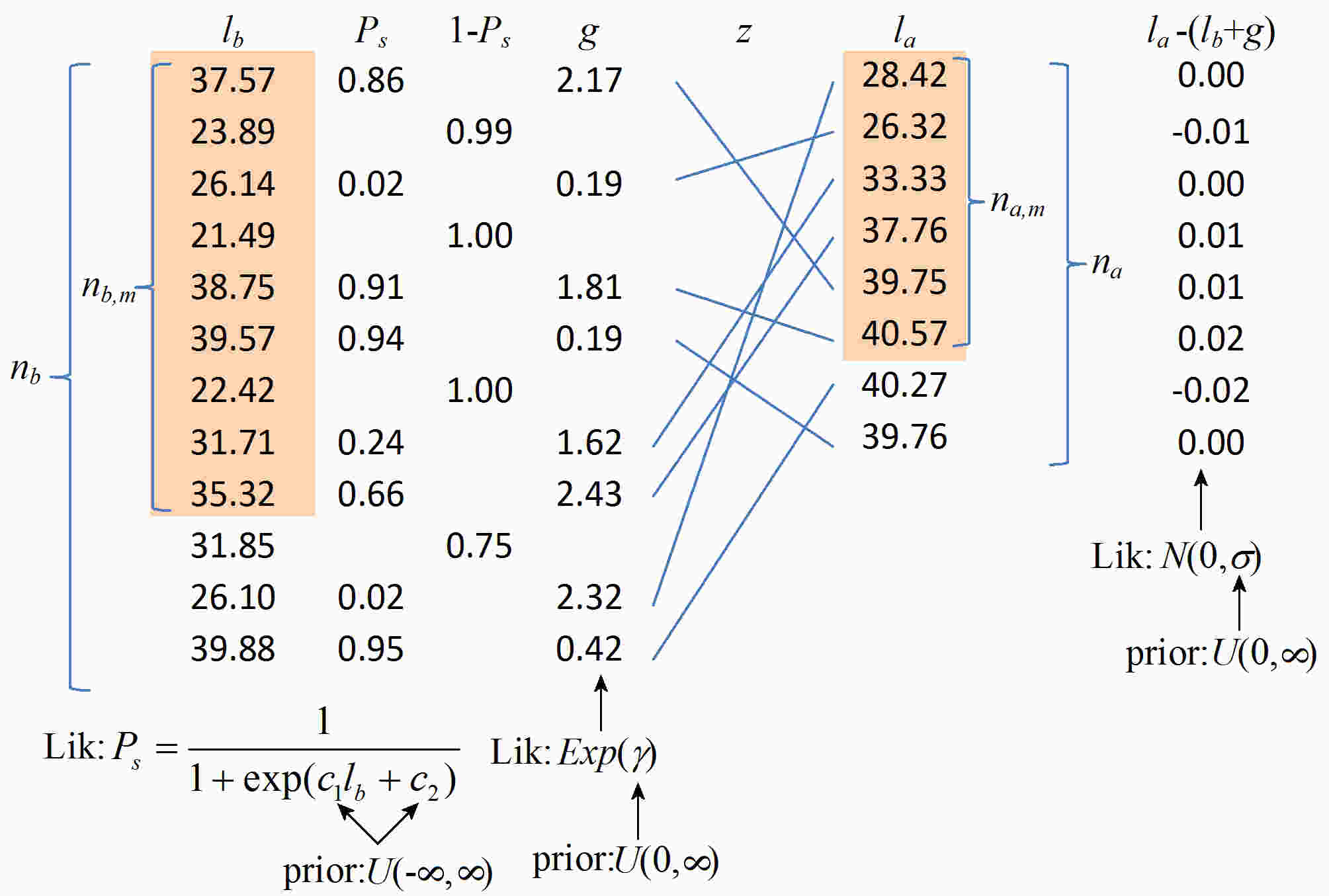


**Fig. S1**: Schematic illustration of the model used to estimate size-dependent winter survival. Symbols are explained in the text. Note that only the shaded values are measured, everything else is inferred. Likelihoods and prior distributions of unknown variables are indicated with “Lik” and “prior”. Priors for *z* (all links equiprobable) and *g* (uniform positive) are not indicated.

As compared to the Bayesian analysis of size-dependent winter mortality by Carlson et al. (2010), our model uses a different mortality function, and does not assume a functional form for the body size distributions before and after winter. In addition, because there is no immigration or emigration from the enclosures, we do not have the problem of distinguishing growth from size-dependent migration, hence our model includes growth. As a final difference, our model includes measurement error.

Initially the individuals that were not measured were assigned lengths that were sampled at random from the lengths of the measured individuals. Subsequently we determined initial values for *z* by minimizing size differences *l_a_*-*l_b_*. (That is, each of the *n_a_* surviving individuals was associated with one of the *n_b_* individuals that closely matched its body length.) The initial values for *g* were then calculated as *l_a_*-*l_b_*, and *γ* as their mean. The standard deviation of measurement error *σ* was initially set to 0.001. Initial values for *c*_1_ and *c*_2_ were obtained by logistic regression of survival (as implied by *z*) on body length *l_b_*. The likelihood of these initial parameter values is given by equations 1-3. (Note that the likelihood of an individual surviving is *P_s_*, and of an individual not surviving is 1-*P_s_*.)

Subsequently, parameter values were estimated by constructing a Monte Carlo Markov Chain using Metropolis and Metropolis-Hastings sampling (Metropolis et al. 1953). Out of 100 000 iterations every 100^th^ was retained, yielding MCMC chains of 1000 samples. Of these, only the last 900 were analysed (excluding the first 100 as burn-in). This was repeated for all 16 ponds separately.

Estimated survival curves are shown, along with body length distributions, in figure S2. For 14 of the 16 enclosures, survival probability increases with body length. MCMC samples from the posterior distributions of parameter *c*_1_ for these 14 enclosures do not include 0, strongly suggesting that survival probability increases with body length. In one enclosure survival probability decreases with body length. Finally, in one enclosure survival appears approximately independent of body length. It should be mentioned that in this enclosure, where survival appeared independent of size, an average growth of *γ* =10 mm was inferred (Fig. S3), which is unrealistically high (Lefebure et al. 2011). Apparently, for this particular enclosure growth and survival cannot be accurately distinguished from the measured size data with the uninformative priors that were used. Indeed, comparison of survival curves (Fig. S2) and growth estimates (Fig. S3) across enclosures reveals that a weak relation between body size and survival is inferred when growth cannot be accurately estimated. In most other enclosures estimates of average growth are around 1-3 mm (Fig. S3). These estimates appear realistic: Temperature was recorded daily in the enclosures, and when we substitute these temperature records in a growth model for the species (Lefebure et al. 2011), we obtain a predicted maximum growth of 2.7 mm between autumn and spring (without resource limitation). Because uninformative priors were used for the unknown parameters (including *γ*), the observation that growth was realistically inferred is most enclosures suggests that the model is appropriate for the data, and that also survival curves were estimated accurately in those enclosures.


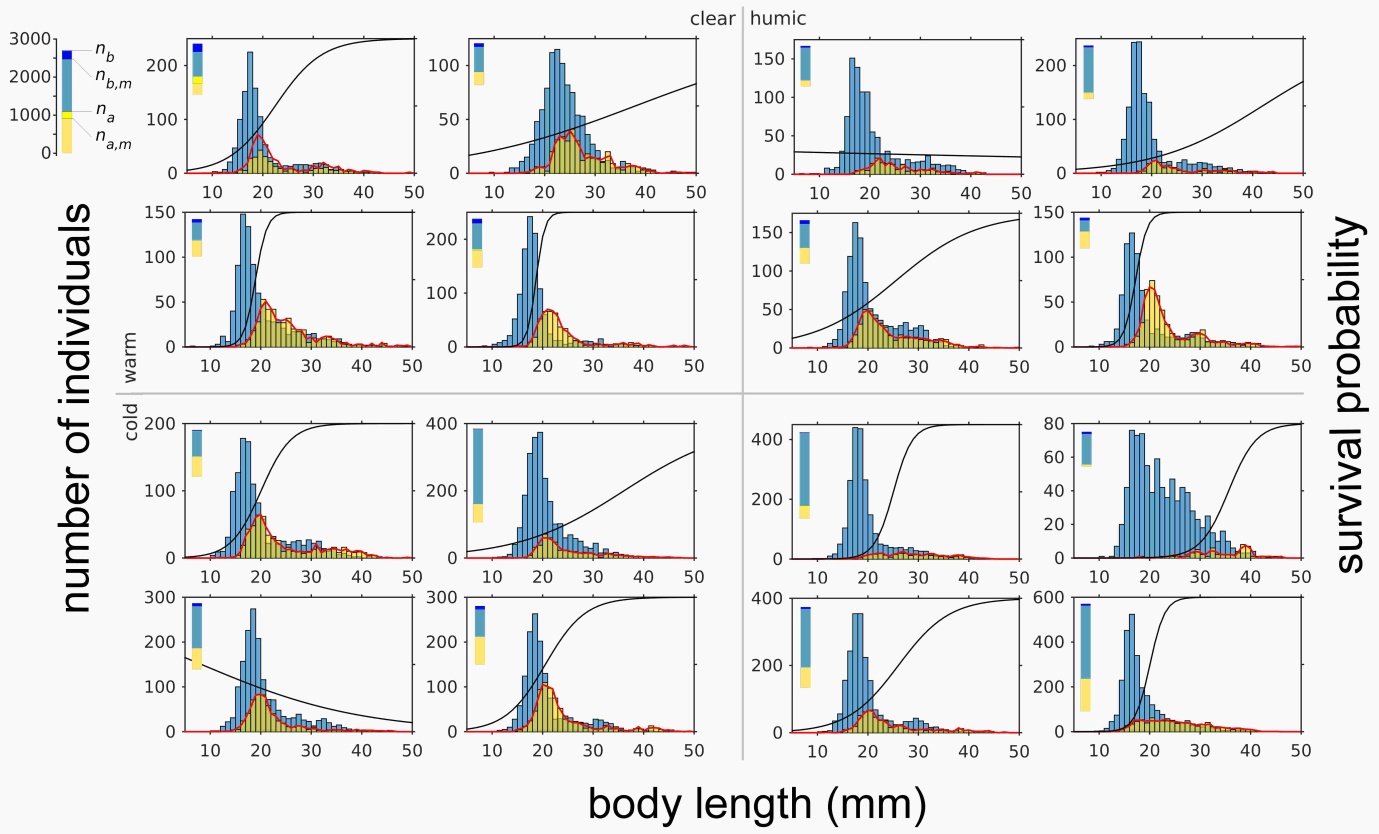


**Fig. S2**: Observed body length distributions before (*l_b_*, *n_b,m_* individuals: blue histogram) and after winter (*l_a_*, *n_a,m_* individuals: yellow histogram), and estimated body size distributions after winter (*l_b_*+*g* averaged over MCMC samples, *n_a_* individuals: red line) for 16 enclosures, grouped by treatment (cold/warm and clear/humic; 4 replicates per treatment). Estimates of the relationship between survival probability (*P_s_*) and body length are indicated with a black line with the vertical axis ranging from 0 to 1. The bar in the upper left corner of each panel indicates population size before (*n_b_*), and after winter (*n_a_*) as well as the number of individuals measured, as illustrated in the left top corner of the figure, where the scale of these bars is also indicated. Note that in most enclosures virtually all individuals were measured before and after winter.


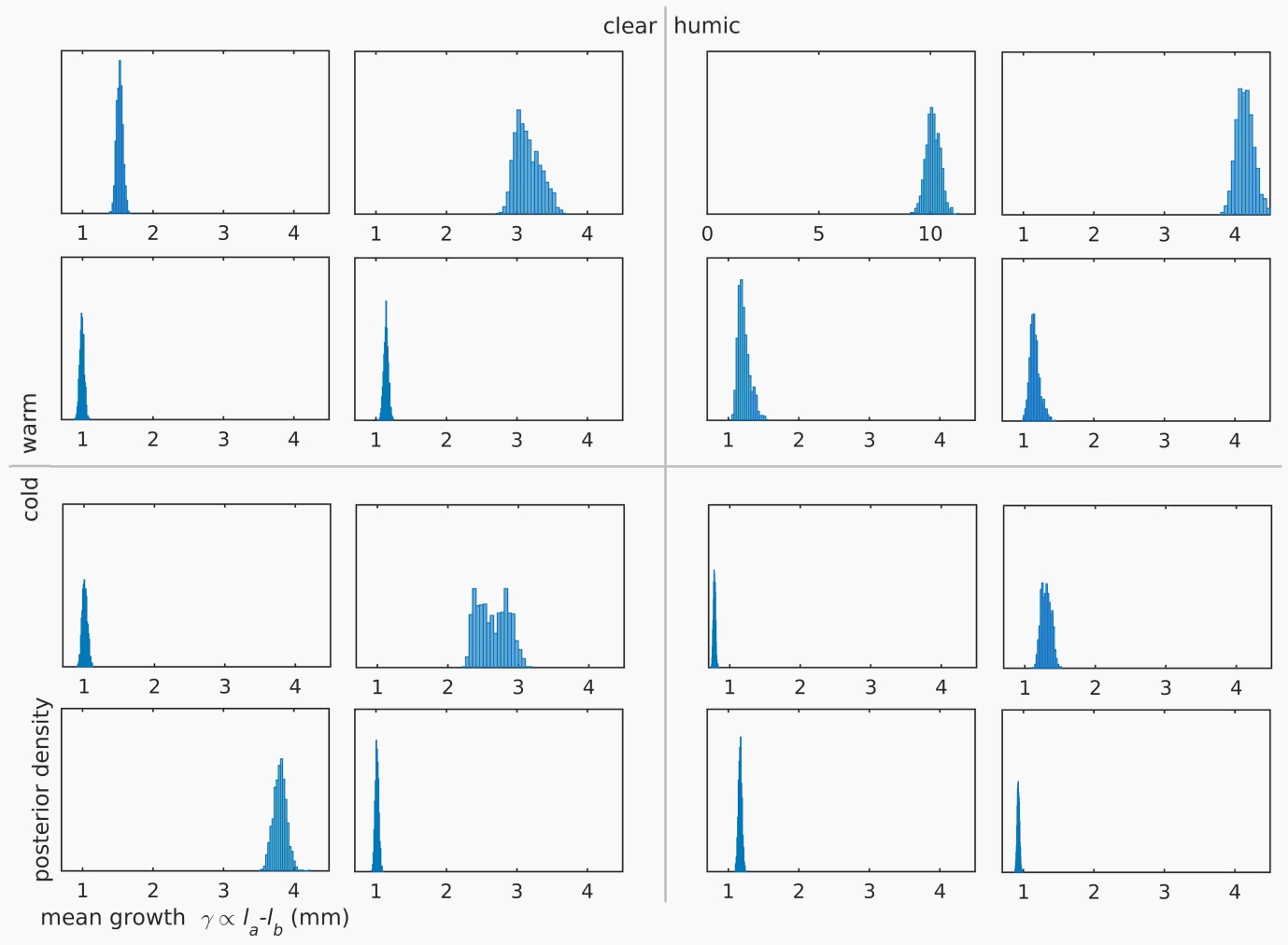


**Fig. S3**: MCMC samples from the posterior distribution of mean growth *γ* for 16 enclosures, grouped by treatment (warm/cold and clear/humic). Note that the third panel on the top row has a different axis scale. Position of panels corresponds to Fig. S2. A temperature-based model of growth (Lefebure et al. 2011) predicts maximum growth of 2.7 mm.

**References**

Carlson, S.M., A.Kottas, and M.Mangel. 2010. Bayesian analysis of size-dependent overwinter mortality from size-frequency distributions. Ecology 9:1016-1024.

Lefebure, R., S. Larsson, and P. Byström. 2011. A temperature-dependent growth model for the three-spined stickleback Gasterosteus aculeatus. Journal of Fish Biology **79**:1815-1827.

Metropolis, N., A.W. Rosenbluth, M.N. Rosenbluth, A.H. Teller, and E.Teller. 1953. Equations of state calculations by fast computing machines. J. Chem. Phys. 21:1087–1092.
