# Supplementary material for: Brownification increases winter mortality in fish
Source: Oecologia. 2016 Dec 3;183(2):587–95. doi: 10.1007/s00442-016-3779-y (PMC5306166; doi:10.1007/s00442-016-3779-y)
Supplement: Supplementary file 2 — Supplementary material 2 (DOCX 44 kb) [file 442_2016_3779_MOESM2_ESM.docx]

BROWNIFICATION INCREASES WINTER MORTALITY IN FISH

Running head: BROWNIFICATION INCREASES WINTER MORTALITY

Electronic Supplementary Material 2

Per Hedström*, David Bystedt, Jan Karlsson, Folmer Bokma and Pär Byström

Department of Ecology and Environmental science, Umeå University, Umeå, SE 90187, Sweden

*Corresponding author: [per.hedstrom@umu.se](mailto:per.hedstrom@umu.se)

Keywords: Brownification, winter mortality, light limitation, feeding efficiency, metabolism

Primary research article

Statistical modelling.

Tables below showing degrees af freedom (DF), F-values, p-values and effect size. Effect size is based on the reduced model. Full models are of the full factorial design of humic*temp*time. Reduced models are minimised from the maximal model by stepwise deletion until only relevant factors and interactions remain. Log Likelihood ratio tests were used to make sure that the reduced models did not differ in degree of explanation of the full model (Pinheiro & Bates, 2006; Crawley, 2007; Bolker *et al.*, 2009; Zuur *et al.*, 2009).

Zooplankton resources

|  | Full model | | | |  | Reduced model | | | |  |  |  |
| --- | --- | --- | --- | --- | --- | --- | --- | --- | --- | --- | --- | --- |
|  | DF | | F | *P* |  | DF | | F | *P* |  |  | Effect size |
| (Intercept) | 1 | 36 | 15.2 | <.001 |  | 1 | 45 | 17.6 | <.0001 |  | Intercept | 0.43 |
| humic | 1 | 12 | <0.01 | 0.95 |  |  |  |  |  |  | Time 2 | -0.32 |
| time | 3 | 36 | 7.5 | <.001 |  | 3 | 45 | 8.45 | <.0001 |  | Time 3 | -0.32 |
| temp | 1 | 12 | 0.69 | 0.42 |  |  |  |  |  |  | Time 4 | -0.39 |
| humic:time | 3 | 36 | 0.98 | 0.41 |  |  |  |  |  |  |  |  |
| humic:temp | 1 | 12 | 0.25 | 0.62 |  |  |  |  |  |  |  |  |
| time:temp | 3 | 36 | 0.11 | 0.95 |  |  |  |  |  |  |  |  |
| humic:time:temp | 3 | 36 | 0.26 | 0.85 |  |  |  |  |  |  |  |  |

Chironomidae resources

|  | Full model | | | |  | Reduced model | | | |  |  |  |
| --- | --- | --- | --- | --- | --- | --- | --- | --- | --- | --- | --- | --- |
|  | DF | | F | *P* |  | DF | | F | *P* |  |  | Effect size |
| (Intercept) | 1 | 36 | 147 | <.0001 |  | 1 | 45 | 144 | <.0001 |  | (Intercept) | 1.88 |
| humic | 1 | 12 | 6.5 | 0.02 |  | 1 | 13 | 6.4 | 0.03 |  | Humic low | -0.74 |
| time | 3 | 36 | 3.2 | 0.04 |  | 3 | 45 | 3.2 | 0.03 |  | Time 2 | 1.03 |
| temp | 1 | 12 | 4.8 | 0.05 |  | 1 | 13 | 4.8 | 0.05 |  | Time 3 | 0.62 |
| humic:time | 3 | 36 | 1.4 | 0.26 |  |  |  |  |  |  | Time 4 | 0.66 |
| humic:temp | 1 | 12 | 2.8 | 0.12 |  |  |  |  |  |  | Temp warm | -0.64 |
| time:temp | 3 | 36 | 0.7 | 0.56 |  |  |  |  |  |  |  |  |
| humic:time:temp | 3 | 36 | 0.4 | 0.75 |  |  |  |  |  |  |  |  |

Total benthic resources

|  | Full model | | | |  | Reduced model | | | |  |  |  |
| --- | --- | --- | --- | --- | --- | --- | --- | --- | --- | --- | --- | --- |
|  | DF | | F | *P* |  | DF | | F | *P* |  |  | Effect size |
| (Intercept) | 1 | 36 | 611 | <.0001 |  | 1 | 45 | 527 | <.0001 |  | Intercept | 1.87 |
| humic | 1 | 12 | 0.5 | 0.47 |  |  |  |  |  |  | Time 2 | 1.06 |
| time | 3 | 36 | 7.5 | <.0001 |  | 3 | 45 | 8.4 | <.01 |  | Time 3 | 0.99 |
| temp | 1 | 12 | 1.6 | 0.20 |  |  |  |  |  |  | Time 4 | 0.69 |
| humic:time | 3 | 36 | 0.6 | 0.61 |  |  |  |  |  |  |  |  |
| humic:temp | 1 | 12 | 3.1 | 0.10 |  |  |  |  |  |  |  |  |
| time:temp | 3 | 36 | 0.9 | 0.44 |  |  |  |  |  |  |  |  |
| humic:time:temp | 3 | 36 | <0.01 | 0.99 |  |  |  |  |  |  |  |  |

Ingested biomass

|  | Full model | | | |  | Reduced model | | | |  |  |  |
| --- | --- | --- | --- | --- | --- | --- | --- | --- | --- | --- | --- | --- |
|  | DF | | F | *P* |  | DF | | F | *P* |  |  | Effect size |
| (Intercept) | 1 | 44 | 116 | <.0001 |  | 1 | 47 | 125 | <.0001 |  | Intercept | 0.93 |
| humic | 1 | 12 | 6.65 | 0.02 |  | 1 | 14 | 7.5 | 0.02 |  | Humic low | 0.28 |
| temp | 1 | 12 | 0.68 | 0.43 |  |  |  |  |  |  |  |  |
| time | 1 | 44 | 13.2 | <.0001 |  | 1 | 47 | 13.3 | <.0001 |  | Time 2 | -0.73 |
| humic:Temp | 1 | 12 | 0.37 | 0.55 |  |  |  |  |  |  | Time 3 | -0.81 |
| humic:time | 1 | 44 | 0.68 | 0.57 |  |  |  |  |  |  | Time 4 | -0.40 |
| temp:time | 1 | 44 | 1.20 | 0.32 |  |  |  |  |  |  |  |  |
| humic:temp:time | 1 | 44 | 0.70 | 0.56 |  |  |  |  |  |  |  |  |

Body condition

|  | Full model | | | |  | | Reduced model | | | |  |  | |  |  |
| --- | --- | --- | --- | --- | --- | --- | --- | --- | --- | --- | --- | --- | --- | --- | --- |
|  | DF | | F | *P* | |  | DF | | F | *P* |  |  | Effect size | |  |
| (Intercept) | 1 | 36 | 8826 | <.0001 | |  | 1 | 45 | 8532 | <.0001 |  | Intercept | 0.78 | |  |
| humic | 1 | 12 | 10.4 | 0.007 | |  | 1 | 14 | 10.0 | 0.01 |  | Humic high | -0.05 | | |
| temp | 1 | 12 | 0.70 | 0.42 | |  |  |  |  |  |  |  |  | | |
| time | 3 | 36 | 24.9 | <.0001 | |  | 3 | 45 | 26.4 | <.0001 |  | Time 2 | -0.12 | | |
| humic:temp | 1 | 12 | 1.78 | 0.21 | |  |  |  |  |  |  | Time 3 | -0.12 | | |
| humic:time | 3 | 36 | 0.34 | 0.80 | |  |  |  |  |  |  | Time 4 | -0.06 | | |
| temp:time | 3 | 36 | 0.86 | 0.47 | |  |  |  |  |  |  |  |  | | |
| humic:temp:time | 3 | 36 | 0.91 | 0.45 | |  |  |  |  |  |  |  |  | | |

Ratio Chironomidae in diet

|  | Full model | | | |  | Reduced model | | | |  |  |  |
| --- | --- | --- | --- | --- | --- | --- | --- | --- | --- | --- | --- | --- |
|  | DF | | F | *P* |  | DF | | F | *P* |  |  | Effect size |
| (Intercept) | 1 | 36 | 60.5 | <.0001 |  | 1 | 45 | 85.0 | <.0001 |  | Intercept | -1.41 |
| humic | 1 | 12 | 1.54 | 0.24 |  |  |  |  |  |  | time2 | -0.64 |
| temp | 1 | 12 | 0.52 | 0.48 |  |  |  |  |  |  | time3 | -2.27 |
| time | 3 | 36 | 2.57 | 0.07 |  | 3 | 45 | 2.79 | 0.05 |  | time4 | -1.29 |
| humic:temp | 1 | 12 | 0.06 | 0.80 |  |  |  |  |  |  |  |  |
| humic:time | 3 | 36 | 0.68 | 0.57 |  |  |  |  |  |  |  |  |
| temp:time | 3 | 36 | 0.89 | 0.45 |  |  |  |  |  |  |  |  |
| humic:temp:time | 3 | 36 | 0.25 | 0.86 |  |  |  |  |  |  |  |  |

Ratio Copepoda in diet

|  | Full model | | | |  | Reduced model | | | |  |  |  |
| --- | --- | --- | --- | --- | --- | --- | --- | --- | --- | --- | --- | --- |
|  | DF | | F | *P* |  | DF | | F | *P* |  |  | Effect size |
| (Intercept) | 1 | 36 | 0.92 | 0.34 |  |  |  | 1.02 | 0.32 |  | Intercept | -2.91 |
| humic | 1 | 12 | 1.16 | 0.30 |  |  |  |  |  |  | time2 | 3.47 |
| temp | 1 | 12 | 0.07 | 0.80 |  |  |  |  |  |  | time3 | 5.61 |
| time | 3 | 36 | 8.86 | <0.001 |  | 1 | 45 | 9.61 | <0.001 |  | time4 | 4.21 |
| humic:temp | 1 | 12 | 0.38 | 0.55 |  |  |  |  |  |  |  |  |
| humic:time | 3 | 36 | 0.47 | 0.70 |  |  |  |  |  |  |  |  |
| temp:time | 3 | 36 | 0.78 | 0.51 |  |  |  |  |  |  |  |  |
| humic:temp:time | 3 | 36 | 0.57 | 0.64 |  |  |  |  |  |  |  |  |

Death rate

|  | Full model | | | |  | Reduced model | | | |  | | |
| --- | --- | --- | --- | --- | --- | --- | --- | --- | --- | --- | --- | --- |
|  | DF | | F | *P* |  | DF | | F | *P* |  |  | Effect size |
| (Intercept) |  |  |  |  |  |  | |  |  |  | Intercept | -0.0049 |
| humic |  |  | 4.21 | 0.06 |  |  | | 4.4 | 0.05 |  | Humic high | -0.0026 |
| temp |  |  | 0.85 | 0.37 |  |  | |  |  |  |  |  |
| humic:temp |  |  | 0.38 | 0.55 |  |  | |  |  |  |  |  |
| residuals | 1 | 12 |  |  |  | 1 | 14 |  |  |  |  |  |

Manova analysis for survival function

|  | Full model | | | Reduced model | | |
| --- | --- | --- | --- | --- | --- | --- |
|  | Df | approx F | Pr(>F) | Df | approx F | Pr(>F) |
| (Intercept) | 1 | 24.74 | <.0001 | 1 | 20.82 | <.0001 |
| Humic | 1 | 0.15 | 0.86 | 1 | 0.17 | 0.85 |
| temp | 1 | 0.25 | 0.79 |  |  |  |
| Humic:temp | 1 | 2.55 | 0.12 |  |  |  |
| Residuals | 12 |  |  | 14 |  |  |

Bolker BM, Brooks ME, Clark CJ, et al (2009) Generalized linear mixed models: a practical guide for ecology and evolution. Trends Ecol Evol 24:127–135. doi: 10.1016/j.tree.2008.10.008

Crawley MJ (2007) The R book. Wiley, Chichester, England ; Hoboken, N.J

Pinheiro J, Bates D (2006) Mixed-effects models in S and S-PLUS. Springer Science & Business Media

Zuur AF, Ieno EN, Walker NJ, et al (2009) Mixed Effects Models and Extensions in Ecology with R. Springer
